# Factorial validity and measurement invariance of the uncertainty response scale

**DOI:** 10.1186/s41155-019-0135-2

**Published:** 2019-12-18

**Authors:** Mariana Lucas Casanova, Lara S. Pacheco, Patrício Costa, Rebecca Lawthom, Joaquim L. Coimbra

**Affiliations:** Universidade do Porto, Faculdade de Psicologia e de Ciências da Educação, Porto, Portugal

**Keywords:** Uncertainty, Coping, Validity, Measurement scales, Invariance

## Abstract

This study presents the adaptation of the Uncertainty Response Scale (Greco & Roger, Pers. Individ. Differ, 31:519-534, 2001) to Portuguese. This instrument was administered to a non-clinical community sample composed of 1596 students and professionals, allowing a thorough validity and invariance analysis by randomly dividing participants into three subsamples to perform: an exploratory factor analysis (sample one: *N* = 512); a preliminary confirmatory factor analysis to identify the final solution for the scale (sample two: *N* = 543); and the confirmatory factor analysis (sample three: *N* = 541). Samples two and three were also used for multi-group analysis to assess measurement invariance, invariance across gender, sociocultural levels, and students versus active professionals. Results showed the scale reflects the original factorial structure, as well as good internal consistency and overall good psychometric qualities. Invariance results across groups reached structural invariance which provides a confident invariance measurement for this scale, while invariance across gender and sociocultural levels reached metric invariance. Accordingly, differences between these groups were explored, by comparing means with multi-group analysis to establish the scale’s sensitivity toward social vulnerability, by demonstrating the existence of statistically significant differences regarding gender and sociocultural levels on how individuals cope with uncertainty, specifically in terms of emotional strategies, as a self-defeating strategy. Thus, females scored higher on emotional uncertainty, as well as low sociocultural levels, compared with higher ones. Therefore, it is proposed that this scale could be a sound alternative to explore strategies for coping with uncertainty, when considering social, economic, or other environmental circumstances that may affect them.

## Introduction

Uncertainty is pervasive to human life throughout space and time. It is present in daily life situations (is it going to rain today?), existential ones (professional and personal decisions), and our relationships with others (loved ones’ illnesses), the world (uncertainty about unemployment during an economic crisis), and nature (fear of natural disasters in a geographical area prone to it). Consequently, the way people cope with uncertainty and uncertain events is of substantial importance. Research in psychology concerning uncertainty began with experimental conditions by authors such as Epstein and Roupenian (1970) and Averill, Olbrich, and Lazarus (1972), exploring its relationship with stress and anxiety. Monat, Averill, and Lazarus (1972) defined uncertainty as the period of anticipation, before confronting a potentially threatening event (or one perceived as such). In these conditions, levels of stress would vary according to people’s efforts to assess and respond toward the event. Later, the concept of intolerance of uncertainty (IU) was defined as the way people understand and process information in situations that can be characterized as uncertain and how they respond with a set of cognitive, emotional and behavioral reactions (Freeston, Rhéaume, Letarte, Dugas, & Ladouceur, 1994).

In this research context, two concepts were used interchangeably for decades (Grenier, Barrette, & Ladouceur, 2005): intolerance of ambiguity (IA) (Frenkel-Brunswik, 1948) and the previously mentioned IU. Some interpretations defined IA as a tendency that individuals may demonstrate to interpret ambiguous situations as a threat and source of discomfort (Kirton, 1981; Majid & Pragasam, 1997; McLain, 1993). According to Bhushan and Amal (1986), ambiguous situations involve novelty, complexity, unpredictability, and uncertainty and people may respond to these situations with a set of reactions: cognitive (rigidity), emotional (discomfort, disapproval, rage or anxiety), and behavioral (rejection or withdrawal). Grenier et al. (2005) analyzed the development of the concept of IU throughout time, highlighting different definitions and how the first definitions of IU were very similar to the ones of IA (Freeston et al., 1994). However, Dugas et al.’ (2005) definition clearly reflects an emotional state that is oriented toward the future, which will allow its distinction from IA, directed to situations of ambiguity that take place in the present (Grenier et al., 2005). More recently, Carleton (2016b) defines IU as “an individual’s dispositional incapacity to endure the aversive response triggered by the perceived absence of salient, key or sufficient information, and sustained by the associated perception of uncertainty” (p. 31). Based on contemporary models involving uncertainty and on research results, Carleton (2012, 2016a, 2016b), proposes that fear of the unknown (defined as a propensity to experience fear caused by the absence of information) may be the fundamental fear from which all fears arise, as well as higher order constructs, such as anxiety sensitivity, which confirms the importance of uncertainty to psychological well-being.

Early results on IU pointed out this construct as having a unique contribution as a predictor of the development of excessive worry (Dugas, Freeston & Ladoucer, 1997; Freeston et al., 1994), as a predictor of worry in daily life, with a lower contribution to worry/generalized anxiety disorder (GAD) than negative metacognitions (Thielsch, Andor, & Ehring, 2015a; Thielsch, Andor, & Ehring, 2015b), presenting a strong association with feelings of worry that could not be explained by other related factors, such as anxiety or depression (Buhr & Dugas, 2002), as well as processes as perfectionism and perceived control (Buhr & Dugas, 2006). Results on the combined effects of fear of anxiety and IU on worry, demonstrated this fear increases in association with IU, enhancing also the levels of worry (Buhr & Dugas, 2009). Various studies demonstrated that people diagnosed with GAD were more intolerant to uncertainty than moderate worriers and individuals with other anxiety disorders (Ladouceur, Gosselin, & Dugas, 2000), which supported the notion of IU being characteristic of worry and GAD. However, further research suggested a relationship between IU and obsessive-compulsive disorder (Gentes & Ruscio, 2011; Holaway, Heimberg, & Coles, 2006; Jacoby, Fabricant, Leonard, Riemann, & Abramowitz, 2013) and social anxiety (Boelen & Reijntjes, 2009; Carleton, Collimore, & Asmundson, 2010; Teale Sapach, Carleton, Mulvogue, Weeks, & Heimberg, 2015), as well as other anxiety-related disorders, such as panic disorder, post-traumatic stress disorder, health anxiety (Boelen, Reijntjes, & Smid, 2015; Carleton, Fetzner, Hackl, & McEvoy, 2013; Fetzner, Horswill, Boelen, & Carleton, 2013; Fetzner et al., 2014; Norton, 2005), but also depression (Hong & Cheung, 2015; McEvoy & Mahoney, 2011). Therefore, recent findings suggest IU is present across diagnostics (Carleton, 2012), which led Thibodeau et al. (2015) to develop scales measuring disorder-specific intolerance of uncertainty, based on the Intolerance of Uncertainty Scale (IUS)—the DSIU.

For this study, the Uncertainty Response Scale (URS) (Greco & Roger, 2001) was chosen to explore individual differences in attitudes toward uncertainty and strategies of coping with uncertainty and not people’s degree of (in)tolerance. This study thus proposes to contribute to the assertion of validity of a psychological measurement scale for the assessment of coping strategies towards uncertainty. Building on research on (in)tolerance of uncertainty, (in)tolerance of ambiguity, and coping strategies, the URS assesses individual differences in coping strategies for uncertainty and to what extent uncertainty is perceived as stress inducing. It is composed of three dimensions: (1) emotional uncertainty as a maladaptive strategy of coping with uncertainty, as an emotional orientation to the problem (correlated with higher levels of neuroticism, reduced self-esteem, emotional rumination, and difficulties to emotionally disconnect before stress inducing situations); (2) cognitive uncertainty, representing coping strategies based on planning and control of uncertainty and so focused on the problem (correlated with tolerance of ambiguity and inversely correlated with social sensitivity, within neuroticism, and with impulsivity); and (3) desire for change, as a positive view of uncertainty and an enjoyment of change (correlated with extraversion, specifically with impulsivity). In its original studies, the scale was assessed through exploratory and confirmatory factor analyses demonstrating good internal consistency and overall psychometric qualities (Greco & Roger, 2001, 2003). Therefore, this scale allows an exploration of emotional coping strategies (that may or may not result in inhibitory behaviors), of cognitive coping strategies (that focus on preparing and planning for the future through a process of reduction of unknowns and, therefore, of reduction of uncertainty), and of a tendency to enjoy change and uncertainty, which could prove useful as another approach to understanding coping with uncertainty.

Through the concept of coping, it is intended to analyze people’s interpretation of uncertainty and the general strategies used to deal with it, not reducing them to a fixed set of resources but considering them as part of the process of giving meaning to uncertainty, considering the living circumstances that surround the individual. Therefore, the personal interpretation of a situation is considered as resulting from the psychological development of the individual and so these strategies may change along personal development (Lazarus & Folkman, 1984). Consequently, coping with a specific situation may entail avoidance, minimization, or acceptance of stress inducing conditions. In this sense, strategies focused on emotional features are used to diminish distress or increase it, allowing to transform the meaning given by the individual to the situation, and, thus, cope with it (as emotional uncertainty may); strategies focused on the problem are directed toward the environment and its transformation or to self-transformation to deal with the situation (as cognitive uncertainty may). Ultimately, we cannot blame the victim for failing to adapt since the main problem relies in the relationship/transaction between people and the social/environmental structures in which they live and that this relationship should be the real target of change (Lazarus & Folkman, 1984).

This paper will thus present results of the process of adaptation of the URS to Portuguese, assessing its factor structure, validity, and reliability through a thorough assessment of three subsamples from a non-clinical community sample of 1596 participants. Multi-group measurement invariance analysis will be assessed, as well as invariance across gender, sociocultural levels, and students versus active professionals (employed or unemployed). To do so, invariance will be tested hierarchically, according to common practice. So, configural invariance will mean the factor structure to be the same across the groups tested, that is, whether similar factors are measured; metric invariance refers to the similarity of factor loadings across groups, besides the previous level of invariance (which allows for comparison of regression slopes); scalar invariance, besides the previous levels of invariance, guarantees that intercepts (latent means) are equivalent across groups, and so factors can be compared; error variance invariance (same factor structure, factor loadings, and error variances), and, finally, structural invariance, which also includes equal factors’ covariance (Vandenberg & Lance, 2000). To the best of our knowledge, no study has ever tested multi-group invariance of the URS, as well as these variables’ invariance. These are particularly important analyses, considering measurement invariance across groups is vital to ensure comparability of scores and to ensure the test measures the same construct, with the same meaning, across groups or cultural variables, such as gender (Vandenberg & Lance, 2000).

So, to demonstrate this scale’s potential and validity, the differences between demographical groups (as indicative of social and economic circumstances) on their responses to URS’ dimensions will be explored, specifically: gender, sociocultural level, and students (from different levels, from technical training to post-graduate students) versus professional participants through multiple-group modeling. It is hypothesized that underprivileged/vulnerable social groups (women, lower sociocultural levels) may present maladaptive strategies to cope with uncertainty, specifically higher levels of emotional uncertainty and, possibly, lower levels of desire for change. In fact, there is evidence in sociological qualitative research that vulnerable social groups, due to increased strain living circumstances, tend to exhibit lower levels of control over uncertainty and may resort to self-defeating strategies to cope with it (Marris, 1996). In this sense, one may consider emotional strategies of coping with uncertainty, as defined in this scale, as self-defeating strategies. Therefore, the final objective of this study is to demonstrate the usefulness of the scale for analyzing coping strategies toward uncertainty, by exploring differences between groups that may adopt different strategies to cope with uncertainty.

Despite the fact that Greco and Roger (2001) only found an effect of gender on systolic blood pressure in a post-task period, women are expected to show higher levels of emotional uncertainty, as it was found in research that focused on similar concepts, with adolescents and adults (Dekkers, Jansen, Salemink, & Huizenga, 2017; Eaton et al., 2012; Koerner & Dugas, 2008), and we can assume they can report lower levels of desire for change, given it implies a positive view on uncertainty.

Accordingly, based on results that show that individuals from lower sociocultural levels are more likely to perceive an environmental threat (economic uncertainty, in these cases) as uncontrollable (Griskevicius et al., 2013; Griskevicius, Delton, Robertson, & Tybur, 2011), it is hypothesized that these groups may also demonstrate difficulties of coping with uncertainty. Furthermore, the authors conclude that this feeling of uncontrollability may lead people from lower sociocultural levels to resort to “fast strategies” (evolutionary strategies focused on reproductive efforts) that prove ultimately ineffective when facing social threats. Therefore, if these groups experience uncertainty as more uncontrollable, they may reveal higher levels of emotional coping and lower levels of desire for change. So, these strategies may be considered self-defeating strategies and, bearing in mind the use of the URS is here proposed to be able to explore differences in coping strategies as influenced by environmental circumstances, testing differences between sociocultural levels seeks to demonstrate its usefulness for exploring other social circumstances.

Since there is no evidence of differences of coping with uncertainty between students and professionals, no differences are expected between these groups. However, considering the added uncertainty that can be found in professional contexts and in the labor market, as well as its psychological effects (de Witte, Pienaar, & de Cuyper, 2016; Giunchi, Emanuel, Chambel, & Ghidlieri, 2016; Jesus et al., 2016; Martín-Artiles, Molina, & Carrasquer, 2016; Mauno, Cheng, & Lim, 2017; Obschonka & Silbereisen, 2015), it was decided to explore if there could be any differences between these two groups. On the other hand, university and technical/professional courses’ students may also feel an added strain of uncertainty, by anticipating the transition to the labor market, which would explain if no differences were found.

In brief, this study has the following aims: (1) to provide evidence for the validity and psychometric properties of the Portuguese version of the URS (testing it through Exploratory Factor Analysis, Confirmatory Factor Analysis, Reliability Assessment, and Multi-Group Invariance); (2) to demonstrate the capacity of the scale to differentiate groups regarding coping strategies toward uncertainty. In order to do so, invariance for gender, sociocultural level, and students versus professionals was tested. Group differences were assessed by comparing means through multi-group models, and it was hypothesized that (i) women may demonstrate higher levels of emotional coping and lower levels of desire for change; individuals from lower sociocultural groups may present higher levels of emotional coping and lower levels of desire for change. Regarding students and professionals, no hypothesis was formulated since there are no previous results in literature that would support for any specific differences. Data was collected online, through a cross-sectional design, resorting to a non-clinical community, convenience sample.

## Methods

### Sample

The complete sample is composed of 1596 participants, from which 55.6% are students and 44.4% are professionals, with an age average of 26.9 (standard deviation (SD) 8.66), and 70.7% females. Regarding sociocultural level distribution (SCL), 36.1% are from middle-lower/lower levels, 19.9% middle level, and 44% middle-upper/upper levels^1^. To perform the analyses intended in this study, this sample was randomly divided in three subsamples, which are composed as described in Table 1. Considering these sample characteristics, the only variable with missing values (m.v.) is age, distributed as following: EFA (sample 1) six m.v.; CFA1 (sample 2) five m.v.; CFA2 (sample 3) nine m.v., in a total of 20 in the complete sample. This study was carried out in accordance with the recommendations and approval of the Ethics Committee of the Faculty of Psychology and Education Sciences of the University of Porto, in Portugal. All participants gave written informed consent in accordance with the Declaration of Helsinki.

**Table 1:** Demographic characteristics by sample (gender, sociocultural levels, students vs. professional, and age) and sample comparison

	Gender		Sociocultural levels (SCL)			Type of participant		Age
	Male	Female	Lower	Middle	Upper	Students	Professionals	
Complete sample (*N* = 1596)	468 (29.3%)	1128 (70.7%)	576 (36.1%)	318 (19.9%)	702 (44.0%)	888 (55.6%)	708 (44.4%)	26.9 (8.61)
EFA (Sample 1) (*N* = 512)	143 (27.9%)	369 (72.1%)	182 (35.5%)	102 (19.9%)	228 (44.5%)	282 (55.1%)	230 (44.9%)	26.8 (8.53)
CFA1 (Sample 2) (*N* = 543)	165 (30.4%)	378 (69.6%)	199 (36.6%)	105 (19.3%)	239 (44%)	309 (56.9%)	234 (43.1%)	27.6 (9.31)
CFA2 (Sample 3) (*N* = 541)	160 (29.6%)	381 (70.4%)	195 (36.0%)	111 (20.5%)	235 (43.4%)	297 (54.9%)	244 (45.1%)	26.3 (7.86)
Sample Comparison χ2 (df)	.79 (2)		.35 (4)			.54 (2)		ANOVA for age: *F*(2, 1573) = 3.3, *p* = .04, *ηp*^2^ = .004 Post-hoc comparisons using Tukey HSD test indicate that the mean score for Sample 2 is significantly different from Sample 3
Sample comparison *p* value	.67		.99			.76		

Gender, sociocultural status, and type of subject characterized by *n* and (%); age characterized as Mean (SD); *χ2* Chi-Square; *df* degrees of freedom, *ANOVA* analysis of variance

### Measures

#### Sociodemographic questionnaire

Includes sociodemographic and situational questions considered pertinent for sample characterization, namely gender, years of schooling, and professional situation. The sociocultural level was calculated based on years of schooling and professional situation of the active professionals, and on years of schooling and professional situation of the parents of students.

#### Uncertainty response scale

Its original version used a scale of four points, but it was decided to change it to a Likert-type scale of five points (between never and always) to increase items’ sensitivity, giving participants more answering options by allowing a midpoint to be available through an odd scale and so, the possibility of a neutral position (Streiner, Norman, & Cairney, 2008). This instrument is composed of 48 items distributed in a three-factor structure: emotional uncertainty with 15 items (reacting to uncertainty with anxiety and sadness, considering it to be a stressor—α = .89 in its original study); cognitive uncertainty with 16 items (as a need to plan, clarify and gather information to reduce uncertainty—α = .85); and desire for change with 17 items (as a feeling of enjoyment and desire towards unexpectedness and change—α = .90) (Greco & Roger, 2001).

For the initial adaptation of the Uncertainty Response Scale (URS) to Portuguese, Casanova, Pacheco, and Coimbra (2010) contemplated linguistic and cultural differences, not only translating but adapting meanings and idiomatic expressions to Portuguese, respecting the contents of the items and its equivalency to assure ecological validity (Casanova, 2010; Casanova, Pacheco & Coimbra, 2010; Casanova & Coimbra, 2011). Two examples of this process of adaptation of expressions would be with the items: «Facing uncertainty is a nerve-wracking experience», which became «*Deparar-me com a incerteza é uma experiência que me* “*dá cabo dos nervos*”», by using a colloquial expression in Portuguese culturally adjusted; and «When making a decision, I am deterred by the fear of making a mistake», which was translated to «*Quando tenho de tomar uma decisão*, *quase paraliso pelo medo de cometer um erro*», to use the metaphor of paralysis, which is quite used within this context in Portuguese. In this process, the authors consulted a group of linguistic experts and of researchers in Psychology, concluding the facial validity analysis with ten interviews with possible participants to guarantee measure equivalency (Tanzer & Sim, 1999; Van de Vijver & Hambleton, 1996).

### Procedures

The sample was collected using an online platform. Several Higher Education Institutions, Training Companies and Centers were contacted, requesting collaboration in the dissemination of the study via email to students and former students, along with the link to the online questionnaire. The email and online questionnaire included a brief explanation of the research and clear, specific, and univocal instructions, while guaranteeing confidentiality and anonymity, allowing abandonment of participation at any moment of the process (Tanzer & Sim, 1999; Van de Vijver & Hambleton, 1996).

**Fig. 1:**
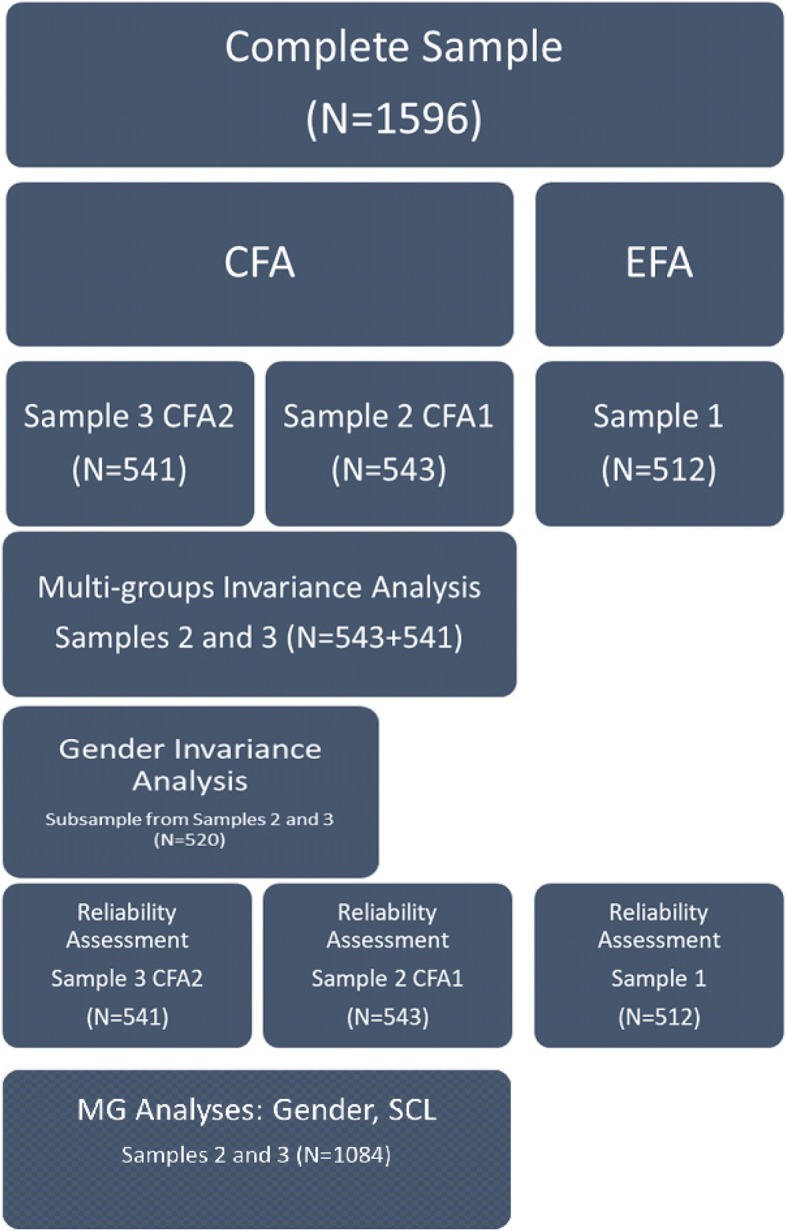
Data analysis procedure and steps

By randomly dividing the sample into three independent samples, the following analyses were performed with the URS: (a) an exploratory factor analysis (sample one); (b) a preliminary confirmatory factor analysis (CFA1 with sample two); (c) a concluding CFA (CFA2 with sample three); (d) reliability analysis (for the three samples, according to the analyses performed with each one). With the samples used in the confirmatory factor analyses (samples two and three), multi-group confirmatory analysis was performed to explore the scale’s invariance (e). Furthermore, a subsample was randomly extracted from both these samples to explore gender invariance with a balanced sample in terms of gender, seeking to maintain the same sample size, around 500 participants. (f) Finally, group differences for gender and sociocultural level were assessed through multi-group analysis, using the samples used for the multi-group confirmatory analysis (g). Figure 1 presents the data analysis procedure and its steps.

Descriptive statistics and exploratory factor analysis were performed using IBM SPSS Statistics 24; confirmatory factor analyses and multi-group analyses were performed using IBM SPSS Amos 24. Thirteen missing values (m.v.) were identified in ten of the URS’ items and so all participants were kept. Statistical analyses using IBM SPSS Statistics 24 were performed excluding missing values cases’ listwise. In confirmatory factor analysis and invariance analyses, m.v. were imputed using regression imputation, according to the CFA’s structure.^2^

CFA was performed to assess if the covariance structure of the model was similar to the covariance structure of the data (Cheung & Rensvold, 2002). Using the maximum likelihood method, the global quality of factorial adjustment was assessed by the main indices and values of reference recommended, assessing its adjustment as proposed by Brown (2006): chi-square test and the chi-square/degrees of freedom between 1 and 2, Comparative Fit Index (CFI) above .90, the root mean square error of approximation (RMSEA), P[RMSEA ≤ 0.05] below .80. The quality of local adjustment was assessed by individual reliability. Therefore, various indices were considered, in view of the sensitivity of the chi-square test to sample size and are here reported (Jackson, Gillaspy, & Purc-stephenson, 2009). Model Expected Cross Validation Index (MECVI) composite measure will be used to compare model fit, being lower scores a demonstration for the goodness of fit and simplicity of one model over the other.

Category 1 invariance was tested through multi-group confirmatory factor analysis, which allows a hierarchical comparison of the unconstrained model with models in which measurement weights, measurement intercepts, structural covariances, and measurement residuals are gradually constricted. The chi-squared difference test was used to assess statistical significance between models. However, considering that differences in chi-square are also dependent on sample size, other criterions were used, namely the Δ CFI, considered when smaller than or equal to − .01 (Cheung & Rensvold, 2002). On the other hand, considering that the number of items and factors affects most adjustment indices, except for RMSEA (Cheung & Rensvold, 2002), Δ RMSEA (considered when < .015), and Δ standardized root mean square residual (SRMR) are also presented, considered when < .025, for loading invariance (Chen, 2007).

Furthermore, category 2 invariance was tested with the groups that proved at least metric invariance, by analyzing between-group differences in latent means, to demonstrate the data’s substantive research interest (Cheung & Rensvold, 2002).

## Results

Descriptive statistics for the individual items of URS were computed to assess its sensitivity (range, means, medians, SD, skewness, and kurtosis) and assert the fulfillment of assumptions for performing exploratory factor analysis and structural equation modeling. Data collected in the three subsamples presents acceptable scores of skewness and kurtosis (Kline, 2005)—highest skewness (sk) and kurtosis (ku) found in items 15 (sk = − 2.14 and ku = 4.55) and 25 (sk = − 1.77 and ku = 4.39) (see Additional file 1: A). A few univariate outliers were identified but were kept in the samples. Preliminary assumption testing was conducted to check for normality, linearity, univariate and multivariate outliers, homogeneity of variance-covariance matrices, and multicollinearity. No serious violations were noted.

### Exploratory factor analysis

Casanova et al. (2010) performed preliminary analysis on this scale, using other extraction and rotation methods (Casanova, 2010; Casanova, Pacheco & Coimbra, 2010; Casanova & Coimbra, 2011). In this study, principal axis factoring (PAF) was used to follow the strategy of the original validation by Greco and Roger (2001), through a reflective model (in contrast with a formative one, which means that in a reflective model, underlying latent causes are what creates effects on indicators and high inter-correlations are expected) (Boorsboom, 2006), with Oblique rotation, since dimensions represent latent variables and were expected to be correlated. In the first analysis, before rotation, most intercorrelations between items proved moderate and low which contributed to the interpretation of unidimensionality in each subscale (Clark & Watson, 1995)—the highest correlation is.68 between items 9 and 13). The anti-image diagonal revealed values above .5 as expected. Bartlett’s sphericity test was significant, but the sample size must be considered given the test’s sensitivity to it and Kaiser-Meyer-Olkin proved satisfactory (.92) (Tabachnick & Fidell, 1996/2007). The scree plot analysis and the values of initial eigenvalues (higher than one) confirmed the three factors solution, which was tested using a conventional exclusion criterion of .40. Seven items were eliminated, as follows: item 33 from the dimension emotional uncertainty; items 18 and 32 from the dimension desire for change; and items 2, 21, 22, and 48 from the dimension cognitive uncertainty, achieving a final solution of 41 items in which items loaded in the expected factors. Table 2 presents factor’s means, SD, and correlations between factors. Additional file 2: B includes factor’s eigenvalues and variance explained, and Additional file 3: C presents the URS distribution with items’ loadings that result from this EFA.

**Table 2:** URS—mean, standard deviation, and correlations between factors (EFA—sample 1)

Factor	Mean	Std. deviation	1	2	3
Emotional uncertainty (1)	41.3	9.71			
Cognitive uncertainty (2)	51.2	6.91	.33**		
Desire for change (3)	53.8	7.84	− .34**	.09	

**p < .001

### Preliminary confirmatory factor analysis (CFA1)

The original study of this scale used item parceling in its CFA given the scale’s length (Greco & Roger, 2001). With the purpose of developing a shorter and robust version of the scale, it was decided not to use this procedure, starting by performing a preliminary CFA for the 41 items, with the second subsample extracted from the complete one. This CFA, thus, assumed an exploratory nature, allowing to examine internal structure validity and to identify items that did not contribute significantly to the model.

In our first analysis, the three factor model of URS revealed low fit (Model A): *X*^2^/df = 3.40, CFI = .80, TLI = .79; RMSEA = .067; P[RMSEA ≤ 0.05] < .001. Additional file 4: D presents the standardized coefficients of CFA1, with the URS distribution after EFA (sample 2)—model A. To achieve a shorter version of the scale and higher reliability, it was decided to retain all items with standardized regression weights above .55 (achieving a practical significance of .31, almost one-third of item variance). So, 16 items were eliminated, as follows: item 1, 44, and 46 from the dimension emotional uncertainty; items 6, 19, 20, 26, 28, 29, and 30, from the dimension cognitive uncertainty; and items 14, 15, 16, 17, 40, and 42, from the dimension desire for change. Considering it was a long scale, and that the final version still is composed of 11 items on emotional uncertainty, six items on cognitive uncertainty, and eight items on desire for change, and bearing in mind the content of the items remaining and of the items eliminated, content validity is believed to have been respected, as it seems to be confirmed by reliability results that will be presented further on. The model then achieved a good measurement fit considering the following indices (Model B1): X2/df = 2.70, CFI = .93, TLI = .92; RMSEA = .056; P[RMSEA ≤ 0.05] = .002. However, modification indices (considering as threshold 11) suggested the correlation between errors of items 9 and 10; items 11 and 13 (within the emotional uncertainty dimension); and items 25 and 26 (within the desire for change dimension). Considering the model modification indices found and the theoretical content shared between these items, it was decided to include these correlations in the final model. The model then achieved the following results (model B2): *X*^2^/df = 2.38, CFI = .94, TLI = .93; RMSEA = .051; P[RMSEA ≤ 0.05] = .422. Comparison between models B1 and B2 through the chi-square difference revealed a significant better fit of model B2, (*X*^2^ (3) = 93, *p* < .001), as well as a lower MECVI (1.66 vs. 1.50), confirming the better fit of model B2. Regarding the chi-square values, it is important to consider the sample size which is commonly accepted to negatively influence models that show a good fit (Bentler, 2007). Additional file 5: E presents the standardized coefficients of this CFA1 with the final URS distribution (sample 2)—model B2.

### Confirmatory factor analysis (CFA2)

In order to confirm the internal structural validity of the scale, another CFA was performed with sample three. Table 3 presents results, comparing the preliminary CFA with the second CFA. The model achieved a good quality of adjustment considering the following indices: *X*^2^/df = 2.49, CFI = .93, TLI = .92; RMSEA = .052; P[RMSEA ≤ 0.05] = .2. Table 4 presents the final solution of the URS and each item’s standardized regression weights. Complete results from this CFA can be found in the Additional file 6: F—CFI above .90, the root mean square error of approximation, RMSEA, P[RMSEA ≤ 0.05] below .80. The quality of local adjustment was assessed by each items’ standardized regression weights. Additional file 7: G presents the Portuguese adaptation of the URS, including all 48 items, highlighting the items that were retained in this final version.

**Table 3:** Goodness of fit indices for the model of the confirmatory factor analyses for the URS with sample 2 (CFA1) and sample 3 (CFA2)

		CFA1 (*N* = 543)								CFA2 (*N* = 541)								
	χ^2^ (df)	*p*	χ^2^/df	CFI	TLI	RMSEA	LO 90	HI 90	PCLOSE	χ^2^ (df)	*p* value	χ^2^/df	CFI	TLI	RMSEA	LO 90	HI 90	PCLOSE
Model A	2636 (776)	*p* < .001	3.40	.80	.79	.067	.064	.069	< .001									
Model B1	734 (272)	*p* < .001	2.70	.93	.92	.056	.051	.061	.02									
Model B2	641 (269)	*p* < .001	2.38	.94	.93	.051	.046	.056	.42	668	*p* < .001	2.49	.93	.92	.052	.047	.057	.20

χ^2^ Chi-square, *df* degrees of freedom, *p p* value, *CFI* comparative fit index, *TLI* Tucker-Lewis Index, *RMSEA* root mean square error of approximation, *LO 90* lower limit of a 90% confidence interval for the population value of RMSEA, *HI 90* upper limit of a 90% confidence interval for the population value of RMSEA, *PCLOSE* RMSEA *p* value

**Table 4:** Distribution URS (final Portuguese version) with original formulations of items, in English—CFA1 (sample 2)

	Items	Standardized regression weights
Emotional uncertainty	4 - Sudden changes make me feel upset.	.73
	5 - When making a decision, I am deterred by the fear of making a mistake.	.65
	8 - When the future is uncertain, I generally expect the worst to happen.	.61
	9 - Facing uncertainty is a nerve-wracking experience.	.80
	10 - I get worried when a situation is uncertain.	.77
	11 - Thinking about uncertainty makes me feel depressed.	.71
	13 - Uncertainty frightens me.	.81
	31 - When I can't clearly discern situations, I get apprehensive.	.60
	35 - When uncertain about what to do next, I tend to feel lost.	.69
	36 - I feel anxious when things are changing.	.62
	41 - When a situation is unclear, it makes me feel angry.	.61
Cognitive uncertainty	3 - I feel better about myself when I know that I have done all I can to accurately plan my future	.60
	7 - I like to have things under control.	.57
	27 - I like to know exactly what I'm going to do next.	.72
	39 - I try to have my life and career clearly mapped out.	.70
	43- I like things to be ordered and in place, both at work and at home.	.61
	47 - I like to plan ahead in detail rather than leaving things to chance.	.74
Desire for change	12 - I find the prospect of change exciting and stimulating.	.64
	23 - I feel curious about new experiences.	.77
	24- I like to think of a new experience in terms of a challenge.	.74
	25 - A new experience is an occasion to learn something new.	.66
	34 - New experiences can be useful.	.69
	37 - New experiences excite me.	.85
	38 - I think variety is the spice of life.	.59
	45- I easily adapt to novelty.	.63

### Reliability

Cronbach alpha coefficients were calculated for the three samples (considering the final confirmatory factor solution) and composite reliability was calculated for the samples used for the CFA1 and CFA2, showing that the scale has satisfactory psychometric properties. To asses convergent validity, factor loadings (standardized regression weights) were used to calculate the average variance extracted (AVE), presenting values that can be considered as acceptable despite the lower values in sample 3—CFA2 (Hair Jr., Anderson, Tatham, & Black, 1998). Therefore, construct validity was supported by factorial validity, supporting item’s specification, and its distribution in the scale’s structure. Discriminant validity of each factor was assessed by comparing each factor’s AVE to the square of correlations between factors. Given that these were inferior to the AVE of the factors involved, discriminant validity was found between all factors. Table 5 presents internal consistency of each factor, the average variance extracted, and composite reliability.

**Table 5:** Construct reliability and validity for the uncertainty response scale (Portuguese version) for the three samples

			Sample 1 (EFA)		Sample 2 (CFA1)			Sample 3 (CFA2)			
Dimensions	α (*n* = 505) (Greco & Roger, 2001)	*N*. items	N. items Portuguese version	α (*n* = 512)	N. items Portuguese final version	α (*n* = 543)	RC	AVE	α (*n* = 541)	RC	AVE
Emotional uncertainty	0.89	15	14	.89	11	.91	.91	.48	.91	.91	.47
Cognitive uncertainty	0.85	17	13	.86	6	.82	.83	.45	.78	.80	.40
Desire for change	0.90	16	14	.87	8	.88	.88	.49	.85	.86	.44
Totals	–	48	41	–	25	–	–	–	–	–	–

α Coefficient Cronbach alpha, *RC* reliability composite, *AVE* average variance extracted

### Multi-group invariance analysis

After reaching the final solution and thereby examining the scale’s internal structure validity, multi-group invariance was analyzed by comparing the samples used for both CFA’s through a series of measurement invariance tests to assess configural invariance by comparing model fit indices of both samples. These results revealed a good fit for groups, proving the same factor structure of the scale in both samples and thus allowing a comparison of domains. Results proved configural invariance of the model: *X*^2^/df = 2.44, CFI = .93, TLI = .93; RMSEA = 0.036; P[RMSEA ≤ .05 > .99]. By analyzing the Δ χ2 test (and respective p value), metric invariance is proved given that factor loadings were found invariant. Moreover, results seem consistent in terms of scalar invariance and error covariances (variance/covariance of residuals found invariant). Finally, covariances of factors were also found invariant and so it is believed that there is sufficient evidence of the scale’s configural, metric, scalar, error variance, and structural invariance, allowing a comparison of regression slopes, factor means, and items’ means. Using the criterion of Δ CFI (Cheung & Rensvold, 2002), the value of Δ CFI for each invariance test confirms the scale’s invariance. Table 6 presents these results in detail.

**Table 6:** Models’ comparison for invariance tests for URS for samples CFA1 and CFA2)

Invariance level	Definition	Model	χ^2^	df	Δ χ^2^	Δ df	*p*	CFI	RMSEA	Δ CFI	Δ RMSEA	Δ SRMR
Configural invariance	Same factor structure	M1	1310.183	538				.93	.036			
Metric invariance	Same factor structure and factor loadings	M2-M1	1329.172	560	18.989	22	.65	.93	.036	0	0	.000
Scalar invariance	Same factor structure, factor loadings, and intercepts	M3-M2	1350.831	585	21.659	25	.66	.93	.035	0	-.001	0
Error variance invariance	Same factor structure, factor loadings, and error variances	M4-M3	1359.107	591	8.275	6	.22	.93	.035	0	0	.002
Structural invariance	Same factor structure, factor loadings, error variances, and factors’ covariance	M5-M4	1391.799	616	32.692	25	.14	.93	.034	0	-.001	.001

χ2 Chi-square, *df* degrees of freedom, Δ χ2 difference between model’s χ2, Δ *df* difference between models’ df, *p p* value, *CFI* comparative fit index, *RMSEA* root mean square error of approximation, Δ *CFI* difference between model’s CFI’s, Δ *RMSEA* difference between model’s RMSEA, Δ *SRMR* difference between model’s standardized root mean square residuals, M1 to M5 models tested

### Multi-group invariance analysis—gender

A subsample was randomly extracted from samples two and three (used for CFA1 and CFA2—see Fig. 1) to achieve a balanced sample for gender invariance assessment. Table 7 presents invariance results for gender, demonstrating an acceptable fit in terms of configural invariance: *X*^2^/df = 1.95, CFI = .90, TLI = .89; RMSEA = .043; P[RMSEA ≤ .05 > .99]. Furthermore, metric invariance was proved through the Δ χ2 test (and respective p value) and Δ CFI. The fact that the Δ RMSEA and the Δ SRMR kept within expected boundaries for structural invariance, as proposed by Chen (2007), gives further support to findings on gender metric invariance.

**Table 7:** Models’ comparison for invariance tests for URS for gender invariance (subsample from samples CFA1 and CFA2: *N* = 520; 268 females; 252 males)

Invariance level	Definition	Model	χ^2^	df	Δ χ^2^	Δ df	*p*	CFI	RMSEA	Δ CFI	Δ RMSEA	Δ SRMR
Configural invariance	Same factor structure	M1	1050.663	538				.90	.043			
Metric invariance	Same factor structure and factor loadings	M2-M1	1077.083	560	26.420	22	.234	.90	.042	− .001	− .001	.002
Scalar invariance	Same factor structure, factor loadings, and intercepts	M3-M2	1167.271	585	90.189	25	< .001	.89	.044	− .012	.002	.000
Error variance invariance	Same factor structure, factor loadings, and error variances	M4-M3	1169.948	591	2.676	6	.848	.89	.043	.001	− .001	.001
Structural invariance	Same factor structure, factor loadings, error variances, and factors’ covariance	M5-M4	1211.157	616	41.209	25	.022	.89	.043	− .003	0	.001

χ^2^ Chi-square, *df* degrees of freedom, Δ χ^2^ difference between model’s χ^2^, Δ *df* difference between models’ df, *p p* value, *CFI* comparative fit index, *RMSEA* root mean square error of approximation, Δ *CFI* difference between model’s CFI’s, Δ *RMSEA* difference between model’s RMSEA, Δ *SRMR* difference between model’s standardized root mean square residuals, M1 to M5 models tested

### Multi-group invariance analysis—sociocultural levels

The samples used for multi-group analysis (samples 2 and 3, used for CFA1 and CFA2—see Fig. 1) were joined to test invariance regarding three groups of sociocultural levels. Table 8 presents results, demonstrating an acceptable fit in terms of configural invariance: *X*^2^/df = 2.13, CFI = .92, TLI = .91; RMSEA = .032; P[RMSEA ≤ .05 > .99]. Moreover, the model achieved metric invariance, by the results of the Δ χ2 test (and respective *p* value), Δ CFI, and Δ RMSEA, although the following levels of invariance were not proven.

**Table 8:** Models’ comparison for invariance tests for URS for sociocultural level invariance (joining samples 2 and 3, *N* = 1084)

Invariance level	Definition	Model	χ^2^	df	Δ χ^2^	Δ df	*p*	CFI	RMSEA	Δ CFI	Δ RMSEA	Δ SRMR
Configural invariance	Same factor structure	M1	1717.985	807				.92	.032			
Metric invariance	Same factor structure and factor loadings	M2-M1	1777.097	851	59.111	44	.064	.92	.032	− .002	0	.001
Scalar invariance	Same factor structure, factor loadings, and intercepts	M3-M2	1876.510	901	99.413	50	< .001	.92	.032	− .004	0	.000
Error variance invariance	Same factor structure, factor loadings, and error variances	M4-M3	1888.243	913	11.734	12	.467	.92	.031	0	− .001	.007
Structural invariance	Same factor structure, factor loadings, error variances, and factors’ covariance	M5-M4	2001.239	969	112.996	56	< .001	.91	.031	− .005	0	.001

χ^2^ Chi-square, *df* degrees of freedom, Δ χ^2^ difference between model’s χ^2^, Δ *df* difference between models’ df, *p p* value, *CFI* comparative fit index, *RMSEA* root mean square error of approximation, Δ *CFI* difference between model’s CFI’s, Δ *RMSEA* difference between model’s RMSEA, Δ *SRMR* difference between model’s standardized root mean square residuals, M1 to M5 models tested

### Multi-group invariance analysis—students and professionals

Regarding the test of multi-group invariance for students versus professionals, the same samples were used. For these groups, acceptable model fit indices were found for configural invariance: *X*^2^/df = 2.50, CFI = .93, TLI = .92; RMSEA = .037; P[RMSEA ≤ .05 > .99]. Metric invariance was not proven, considering the *p* value of the Δ χ2 test, although results for Δ CFI, and Δ RMSEA, fall within accepted boundaries for metric invariance, as can be analyzed in Table 9. Therefore, for caution purposes, it was decided not to test differences between these groups.

**Table 9:** Models’ comparison for invariance tests for URS for students and professionals invariance (joining samples 2 and 3; *N* = 1084)

Invariance level	Definition	Model	χ^2^	df	Δ χ^2^	Δ df	*p*	CFI	RMSEA	Δ CFI	Δ RMSEA	Δ SRMR
Configural invariance	Same factor structure	M1	1345.358	538				.93	.037			
Metric invariance	Same factor structure and factor loadings	M2-M1	1388.303	560	42.946	22	.005	.93	.037	− .002	0	.001
Scalar invariance	Same factor structure, factor loadings, and intercepts	M3-M2	1483.317	585	95.013	25	< .001	.92	.038	− .006	.001	.000
Error variance invariance	Same factor structure, factor loadings, and error variances	M4-M3	1486.278	591	2.962	6	.814	.92	.037	0	− .001	.000
Structural invariance	Same factor structure, factor loadings, error variances, and factors’ covariance	M5-M4	1576.363	619	90.085	28	< .001	.92	.038	− .005	.001	.000

χ^2^ Chi-square, *df* degrees of freedom, Δ χ^2^ difference between model’s χ^2^, Δ *df* difference between models’ df, *p p* value, *CFI* comparative fit index, *RMSEA* root mean square error of approximation, Δ *CFI* difference between model’s CFI’s, Δ *RMSEA* difference between model’s RMSEA, Δ *SRMR* difference between model’s standardized root mean square residuals, M1 to M5 models tested

### Gender and SCL group differences using multi-group analysis

In order to explore the scale’s sensitivity, multiple-group analyses were performed on gender and sociocultural level, joining samples 2 and 3, to compare means between these groups. It was hypothesized that women and individuals from lower sociocultural backgrounds could present maladaptive strategies to cope with uncertainty, specifically higher levels of emotional uncertainty and lower levels of desire for change. So, the means of the latent factors of emotional uncertainty, coping with uncertainty, and desire for change in males and females were analyzed through a series of restricted hierarchical models that were compared to a model of means’ equality, by using males as a reference group. All *p* values were calculated using the Bonferroni correction to avoid type I errors. Through this procedure, statistically significant differences were found for emotional uncertainty, using a *Z* test of critical ratios, providing evidence for the hypothesis that women present higher levels of emotional uncertainty than men (*B* = .33; *p* < .05). No differences were found for cognitive coping, while differences for desire for change proved non-significant. Therefore, our hypothesis was partly confirmed.

Regarding sociocultural levels, the same procedures were followed. However, considering this variable is composed of three groups, a step-by-step analysis was performed, by comparing lower levels with middle or higher levels; middle levels with lower or higher ones; and higher levels with the lower or middle levels. No statistically significant differences were found between middle levels and the other ones, and between higher levels and the other ones, for any of the dimensions. Statistically significant differences were found through a Z test of critical ratios between lower and middle or higher sociocultural levels, showing that lower sociocultural levels revealed more emotional uncertainty (*B* = .16; *p* < .05) than the other sociocultural levels. Furthermore, lower sociocultural levels showed lower levels of desire for change than middle or higher sociocultural levels (*B* = − .079) but these differences did not remain significant, using the Bonferroni correction. There were no significant differences for cognitive uncertainty for these groups.

## Discussion

This study aimed to contribute to the development of a psychological measure in Portuguese for the assessment of strategies of coping with uncertainty, focusing on the scale’ factor structure, validity and reliability, as well as group invariance and invariance across gender, sociocultural levels, and students versus active professionals, concluding with an exploration of the scale’s sensitivity to demographical variables, searching for group differences. Concerning these demographical variables, it was expected that women and individuals from lower SCL would demonstrate higher levels of emotional uncertainty and lower levels of desire for change. No differences were expected between types of participants—students and active professionals.

Validation results demonstrate the process of adaptation respected the original scale since all items loaded within their expected factors, supporting construct validity. The strategy of performing a preliminary CFA proved useful since it allowed to reduce the number of items, while maintaining its structure and psychometric qualities, reaching a good adjustment quality, which was confirmed by the second CFA performed. The scale proved to be reliable and valid, with very good to excellent internal consistency values and good composite reliability levels, contributing to a sense of “global quality,” as proposed by Borsboom, Mellenbergh, and Heerder (2004). Furthermore, divergent reliability results, assessed by AVE, reached acceptable levels.

Multi-group measurement invariance of the scale reached very good results across groups, proving structural invariance of the scale, which renders psychometric support to the comparability of cross-sectional studies using the URS. Invariance across gender and sociocultural levels reached metric invariance, which allows for the comparison of regression slopes. However, comparability of these results must be assessed with caution and so, invariance among these groups should be verified by further studies. Furthermore, only configural invariance was proved between students and professionals, which may indicate disagreement on how the constructs manifest for these groups. Consequently, group differences using multi-group analysis were not performed between students and professionals. Nevertheless, it was decided to explore group differences through multi-group analysis for gender and SCL, considering their results of Δ RMSEA and Δ SRMR for scalar invariance, as proposed by Chen (2007), as well as the Δ CFI for SCL in the assessment of scalar invariance (Cheung & Rensvold, 2002).

Concerning the results of group differences through multi-group analyses, an effect of gender was found, specifically within emotional uncertainty, in which women obtained higher scores, as previously found in researches on IU, in the inhibitory IU subscale (Dekkers et al., 2017), which provides additional support for the scale’s sensitivity across gender. In addition, differences were found, as hypothesized, between different SCL in terms of emotional uncertainty. These results support the scale’s definition of emotional uncertainty as a maladaptive strategy to cope with uncertainty, what can be interpreted as a self-defeating strategy to which people may resort when faced with challenges felt as overwhelming and uncontrollable, that may reinforce conditions of vulnerability. These results are supported by previous research that demonstrates that, as fear of the unknown increases, people prefer to accept its negative consequences than to tolerate uncertainty (Buhr & Dugas, 2002; Ghosh & Ray, 1997; Rassin & Muris, 2005), as well as results that demonstrated that people from lower SCL, when facing uncertainty, could resort to strategies that may prove inefficient (Griskevicius et al., 2013; Griskevicius, Delton, Robertson, & Tybur, 2011). No significant differences were found between these groups (gender and SCL) for the dimension desire for change, as in the original study for gender (Greco & Roger, 2001), which may mean that this variable does not differentiate social groups and so, that groups that experience greater social security or stability, as men or individuals from higher SCL, do not necessarily reveal a higher desire for change.

The existence of significant differences for gender and SCL levels regarding emotional uncertainty supports the scale’s sensitivity to distinguish groups, which supports its criterion and concurrent validity. These results are reinforced by previous findings with similar constructs, such as the ones of Koerner and Dugas (2008), in which being a female predicted negative appraisals of ambiguous scenarios. Even though other studies show no gender effects on IUS (Allan, Oglesby, Uhl, & Schmidt, 2016), others found that gender could be a predictor of implicit memory for uncertain and neutral words but not necessarily IU (Francis, Dugas, & Ricard, 2016), and others found significant gender effects in the contrary direction (adolescent boys scored higher on IU)—in this particular case, cultural differences could be the origin of these results (Barahmand, 2008). Nevertheless, these results may be explained by the fact that IUS focuses on individual vulnerabilities, in which there may be no effects of gender, while URS, by focusing on coping strategies, may allow to identify an effect of gender in resorting to such self-defeating strategies, when people face greater environmental strain. This is supported by results on the predictive capacity of emotion regulation strategies for anxiety and worry and its differences in terms of gender (Zlomke & Hahn, 2010).

No effects of gender or SCL were found on cognitive uncertainty as expected. This was expected given empirical results that demonstrate that only an emotional orientation to problems (compared to a cognitive or behavioral one) contributed to the prediction of worry in its relationship to intolerance of uncertainty (Dugas, Freeston, & Ladouceur, 1997). Moreover, considering the parallel established between cognitive uncertainty and prospective IU subscale, in Dekkers et al.’s (2017) findings, there were also no gender differences in prospective IU.

In terms of limitations, this study is based on a convenience sample, with unbalanced groups, and so, these results could be supported by further studies, namely, of invariance across gender, sociocultural levels, and different groups from the general population. A possibility is to further explore the effect of SCL in a more balanced sample and to explore the relationship of coping with uncertainty with other significant concepts, which would give further evidence of the scale’s convergent and concurrent validity, which were assessed with positive results in the original studies of Greco and Roger (2001, 2003). Moreover, it would be useful to explore its longitudinal invariance and predictive power through longitudinal studies. Despite these limitations, results attest to the scale’s value and that it could make a meaningful contribution in research by allowing an additional perspective for the examination of uncertainty, as a complement to the construct of IU, already greatly investigated.

## Conclusions

To sum up, regardless of the reduction of the number of items, the scale provides a psychometrically sound assessment of coping strategies toward uncertainty in a shorter version, which may be an advantage in future applications. Furthermore, this scale may prove useful for the understanding of coping strategies toward uncertainty, expanding possibilities of research on uncertainty on community samples. Given that fear of the unknown, as defined by Carleton (2012, 2016a, 2016b) may be considered as the fundamental fear, and as an evolutionary and adaptive one, we can reason that despite different levels of (in)tolerance of uncertainty, all people experience psychological challenges in coping with uncertainty. Furthermore, since fear of the unknown “…may encompass external environmental uncertainties and threats and an individual’s internally oriented uncertainty about his or her own resources to deal with such threats” (Hong & Cheung, 2015; p.904), URS may allow for an approximation, along with other psychological measures, to analyzing this interaction between environmental uncertainties and perception on internal resources, which are affected by individual and social circumstances. Consequently, building on initial results found in this study on demographical variables, it would be relevant to further explore if coping strategies vary according to other dimensions of social vulnerability and living contexts (such as unemployment or underemployment, socioeconomic levels, ethnic backgrounds, schooling levels, among others that may characterize impoverished or in danger communities), which would prove useful for a psychosocial take on uncertainty through quantitative methodologies.

## Supplementary information

**Additional file 1.** A. Descriptive statistics for the URS (Portuguese Version)

**Additional file 2.** B. URS’s Exploratory Factor Analysis – eigenvalues, variance explained

**Additional file 3.** C. EFA - PAF with Oblique rotation (.4) – URS Distribution with item loadings

**Additional file 4.** D. CFA1 - URS Distribution after EFA (Sample 2); standardized coefficients (Model A)

**Additional file 5.** E. CFA1 - Final URS Distribution (Sample 2); standardized coefficients (Model B2)

**Additional file 6.** F. CFA2 - Final URS Distribution (Sample 3); standardized coefficients (Model B2)

**Additional file 7.** G. Portuguese Adaptation of the Uncertainty Response Scale (48 items)

## Data Availability

The raw data supporting the conclusions of this manuscript will be made available by the authors, without undue reservation, to any qualified researcher.

## Abbreviations

ANOVA: Analysis of variance
AVE: Average variance extracted
CFA: Confirmatory factor analysis
CFI: Comparative Fit Index
df: Degrees of freedom
DSIU: Disorder-specific intolerance of uncertainty
EFA: Exploratory factor analysis
GAD: Generalized anxiety disorder
IA: Intolerance of ambiguity
IU: Intolerance of uncertainty
IUS: Intolerance of uncertainty scale
Ku: Kurtosis
m.v.: Missing values
MECVI: Model Expected Cross Validation Index
p: *P* value
PAF: Principal axis factoring
RMSEA: Root mean square error of approximation
SCL: Sociocultural level
Sk: Skewness
SRMR: Standardized root mean square residual
TLI: Tucker-Lewis Index
URS: Uncertainty Response Scale
χ2: Chi-square

## Footnotes

1 Most participants were identified as middle and upper levels of SCL since this variable was defined by mostly considering educational levels and so this is not considered a measure of socio-economic levels. Furthermore, the form of data collection (online) constrains access to other segments of population.

2 Distribution of missing values (m.v.) per item: 1 m.v. on items 5, 8, 10, 11, 13, 28, 46; 2 m.v. on items 7, 15, 42. These m.v. were randomly distributed by the three samples: 7 items in the EFA Sample; 4 items in the CFA1 Sample; 2 items in the CFA2 Sample. Considering these were imputed for the CFA’s, they do not impact the following analyses, just impacting the EFA sample and the analyses related to it.

## References

[CR1] Allan, N. P., Oglesby, M. E., Uhl, A., & Schmidt, N. B. (2016). Cognitive risk factors explain the relations between neuroticism and social anxiety for males and females. *Cognitive Behaviour Therapy*, 6073(March), 1–15. http://doi.org/10.1080/16506073.2016.1238503 10.1080/16506073.2016.1238503 27690746

[CR2] Averill JR, Olbrich E, Lazarus RS (1972). Personality correlates of differential responsiveness to direct and vicarious threat: a failure to replicate previous findings. Journal of personality and social psychology..

[CR3] Barahmand U (2008). Age and gender differences in adolescent worry. Personality and Individual Differences.

[CR4] Bhushan LI, Amal SB (1986). A situational test of intolerance of ambiguity. Psychologia: An International Journal of Psychology in the Orient.

[CR5] Boelen Paul A., Reijntjes Albert (2009). Intolerance of uncertainty and social anxiety. Journal of Anxiety Disorders.

[CR6] Boelen PA, Reijntjes A, Smid GE (2015). Concurrent and prospective associations of intolerance of uncertainty with symptoms of prolonged grief, posttraumatic stress, and depression after bereavement. Journal of Anxiety Disorders.

[CR7] Borsboom Denny (2006). The attack of the psychometricians. Psychometrika.

[CR8] Borsboom Denny, Mellenbergh Gideon J., van Heerden Jaap (2004). The Concept of Validity. Psychological Review.

[CR9] Brown, T. A. (2006). *Confirmatory factor analysis for applied research*. New York, NY: Guilford Press.

[CR10] Buhr K, Dugas MJ (2002). The intolerance of uncertainty scale: Psychometric properties of the English version. Behaviour Research and Therapy.

[CR11] Buhr Kristin, Dugas Michel J. (2006). Investigating the construct validity of intolerance of uncertainty and its unique relationship with worry. Journal of Anxiety Disorders.

[CR12] Buhr Kristin, Dugas Michel J. (2009). The role of fear of anxiety and intolerance of uncertainty in worry: An experimental manipulation. Behaviour Research and Therapy.

[CR13] Carleton R. Nicholas, Collimore Kelsey C., Asmundson Gordon J.G. (2010). “It's not just the judgements—It's that I don’t know”: Intolerance of uncertainty as a predictor of social anxiety. Journal of Anxiety Disorders.

[CR14] Carleton R Nicholas (2012). The intolerance of uncertainty construct in the context of anxiety disorders: theoretical and practical perspectives. Expert Review of Neurotherapeutics.

[CR15] Carleton R. Nicholas (2016). Fear of the unknown: One fear to rule them all?. Journal of Anxiety Disorders.

[CR16] Carleton R. Nicholas (2016). Into the unknown: A review and synthesis of contemporary models involving uncertainty. Journal of Anxiety Disorders.

[CR17] Carleton R. Nicholas, Fetzner Mathew G., Hackl Jennifer L., McEvoy Peter (2013). Intolerance of Uncertainty as a Contributor to Fear and Avoidance Symptoms of Panic Attacks. Cognitive Behaviour Therapy.

[CR18] Casanova, M., Pacheco, L., & Coimbra, J.L. (2010). New forms of uncertainty in the individualized society: Adaptation and validation of the uncertainty response scale (URS, Greco & Roger, 2001) to Portuguese Population and the Creation of a Scale on the Perception of Uncertainty in the Social Context and its Psychological Consequences. *Proceedings: “Competing Values in an Uncertain Environment: Managing the Paradox - International Society for the Study of Work and Organizational Values”*. Estoril: ISSWOV.

[CR19] Casanova, M. L (2010). Dimensões Histórico-sociais da Incerteza Psicológica: Contributos Metodológicos e a Construção de um Instrumento Original. [Sociohistorical Dimensions of Psychological Uncertainty: Methodological Contributions and the Construction of an Original Instrument]. (master thesis). Porto: Universidade do Porto.

[CR20] Casanova, ML. & Coimbra, J.L. (2011). Dimensões Histórico-sociais da Incerteza Psicológica e o Desenvolvimento Vocacional / Profissional: Contributos Metodológicos e um Instrumento Original. [Sociohistorical Dimensions of Psychological Uncertainty and Vocational/Professional Development: Methodological Contributions and an Original Instrument]. Livro de Actas: XI Congreso Internacional Galego-Portugués de Psicopedagoxía na Universidade da Coruña. A Coruña.

[CR21] Chen Fang Fang (2007). Sensitivity of Goodness of Fit Indexes to Lack of Measurement Invariance. Structural Equation Modeling: A Multidisciplinary Journal.

[CR22] Cheung, G. W., & Rensvold, R. B. (2002). Evaluating goodness-of-fit indexes for testing measurement invariance. *Structural Equation Modeling*, 9(2), 233–255. http://doi.org/10.1207/S15328007SEM0902

[CR23] Clark LA, Watson D (1995). Constructing validity: Basic issues in objective scale Development. Psychological Assessment.

[CR24] de Witte H, Pienaar J, de Cuyper N (2016). Review of 30 years of longitudinal studies on the association between job insecurity and health and well-being: Is there causal evidence?. Australian Psychologist.

[CR25] Dekkers Laura M.S., Jansen Brenda R.J., Salemink Elske, Huizenga Hilde M. (2017). Intolerance of Uncertainty Scale: Measurement invariance among adolescent boys and girls and relationships with anxiety and risk taking. Journal of Behavior Therapy and Experimental Psychiatry.

[CR26] Dugas Michel J., Freeston Mark H., Ladouceur Robert (1997). Cognitive Therapy and Research.

[CR27] Dugas Michel J., Hedayati Mary, Karavidas Angie, Buhr Kristin, Francis Kylie, Phillips Natalie A. (2005). Intolerance of Uncertainty and Information Processing: Evidence of Biased Recall and Interpretations. Cognitive Therapy and Research.

[CR28] Eaton Nicholas R., Keyes Katherine M., Krueger Robert F., Balsis Steve, Skodol Andrew E., Markon Kristian E., Grant Bridget F., Hasin Deborah S. (2012). An invariant dimensional liability model of gender differences in mental disorder prevalence: Evidence from a national sample. Journal of Abnormal Psychology.

[CR29] Epstein S, Roupenian A (1970). Heart rate and skin conductance during experimentally induced anxiety: The effects of anticipated intensity of noxious stimulation and experience. Journal of Experimental Psychology.

[CR30] Fetzner Mathew G., Asmundson Gordon J.G., Carey Cori, Thibodeau Michel A., Brandt Chad, Zvolensky Michael J., Carleton R. Nicholas (2014). How Do Elements of a Reduced Capacity to Withstand Uncertainty Relate to the Severity of Health Anxiety?. Cognitive Behaviour Therapy.

[CR31] Fetzner Mathew G., Horswill Samantha C., Boelen Paul A., Carleton R. Nicholas (2013). Intolerance of Uncertainty and PTSD Symptoms: Exploring the Construct Relationship in a Community Sample with a Heterogeneous Trauma History. Cognitive Therapy and Research.

[CR32] Francis Kylie, Dugas Michel J., Ricard Nathalie C. (2016). An exploration of Intolerance of Uncertainty and memory bias. Journal of Behavior Therapy and Experimental Psychiatry.

[CR33] Freeston Mark H., Rhéaume Josée, Letarte Hélène, Dugas Michel J., Ladouceur Robert (1994). Why do people worry?. Personality and Individual Differences.

[CR34] FRENKEL-BRUNSWIK ELSE (1949). INTOLERANCE OF AMBIGUITY AS AN EMOTIONAL AND PERCEPTUAL PERSONALITY VARIABLE. Journal of Personality.

[CR35] Gentes Emily L., Ruscio Ayelet Meron (2011). A meta-analysis of the relation of intolerance of uncertainty to symptoms of generalized anxiety disorder, major depressive disorder, and obsessive–compulsive disorder. Clinical Psychology Review.

[CR36] Ghosh Dipankar, Ray Manash R. (1997). Risk, Ambiguity, and Decision Choice: Some Additional Evidence. Decision Sciences.

[CR37] Giunchi Marianna, Emanuel Federica, Chambel Maria José, Ghislieri Chiara (2016). Job insecurity, workload and job exhaustion in temporary agency workers (TAWs). Career Development International.

[CR38] Greco Veronica, Roger Derek (2001). Coping with uncertainty: the construction and validation of a new measure. Personality and Individual Differences.

[CR39] Greco Veronica, Roger Derek (2003). Uncertainty, stress, and health. Personality and Individual Differences.

[CR40] Grenier S, Barrette AM, Ladouceur R (2005). Intolerance of Uncertainty and Intolerance of Ambiguity: Similarities and differences. Personality and Individual Differences.

[CR41] Griskevicius V, Ackerman JM, Cantu SM, Delton AW, Robertson TE, Simpson JA (2013). When the economy falters, do people spend or save? Responses to resource scarcity depend on childhood environments. Psychological Science.

[CR42] Griskevicius Vladas, Delton Andrew W., Robertson Theresa E., Tybur Joshua M. (2011). Environmental contingency in life history strategies: The influence of mortality and socioeconomic status on reproductive timing. Journal of Personality and Social Psychology.

[CR43] Hair JF, Anderson RE, Tatham RL, Black WC (1998). Multivariate data analysis.

[CR44] Holaway Robert M., Heimberg Richard G., Coles Meredith E. (2006). A comparison of intolerance of uncertainty in analogue obsessive-compulsive disorder and generalized anxiety disorder. Journal of Anxiety Disorders.

[CR45] Hong Ryan Y., Cheung Mike W.-L. (2014). The Structure of Cognitive Vulnerabilities to Depression and Anxiety. Clinical Psychological Science.

[CR46] Jackson DL, Gillaspy JA, Purc-stephenson R (2009). Reporting practices in confirmatory factor analysis. An overview and some recommendations.

[CR47] Jacoby Ryan J., Fabricant Laura E., Leonard Rachel C., Riemann Bradley C., Abramowitz Jonathan S. (2013). Just to be certain: Confirming the factor structure of the Intolerance of Uncertainty Scale in patients with obsessive-compulsive disorder. Journal of Anxiety Disorders.

[CR48] Jesus SN, Leal AR, Viseu JN, Valle P, Matavelli RD, Pereira J, Greenglass E (2016). Coping as a moderator of the influence of economic stressors on psychological health. Analise Psicologica.

[CR49] Kirton M.J. (1981). A Reanalysis of Two Scales of Tolerance of Ambiguity. Journal of Personality Assessment.

[CR50] Kline RB (2005). *Principle and practice of structural equation modelling*.

[CR51] Koerner Naomi, Dugas Michel J. (2007). An investigation of appraisals in individuals vulnerable to excessive worry: the role of intolerance of uncertainty. Cognitive Therapy and Research.

[CR52] Ladouceur Robert, Gosselin Patrick, Dugas Michel J (2000). Experimental manipulation of intolerance of uncertainty: a study of a theoretical model of worry. Behaviour Research and Therapy.

[CR53] Lazarus RS, Folkman S (1984). *Stress, appraisal and coping*.

[CR54] Majid Abdul, Pragasam John (1997). Interactions of Intolerance of Ambiguity and of Contingent Liability on Auditors' Avoidance of Litigation. Psychological Reports.

[CR55] Marris P (1996). The politics of uncertainty: Attachment in private and public life.

[CR56] Martín-Artiles A, Molina O, Carrasquer P (2016). Incertidumbre y actitudes pro-redistributivas : mercados de trabajo y modelos de bienestar en. Europa Uncertainty and Attitudes Pro-redistributive : Política y Sociedad.

[CR57] Mauno Saija, Cheng Ting, Lim Vivian (2017). The Far-Reaching Consequences of Job Insecurity: A Review on Family-Related Outcomes. Marriage & Family Review.

[CR58] McEvoy PM, Mahoney AEJ (2011). Achieving certainty about the structure of intolerance of uncertainty in a treatment-seeking sample with anxiety and depression. Journal of Anxiety Disorders.

[CR59] Mclain David L. (1993). The Mstat-I: A New Measure of an Individual'S Tolerance for Ambiguity. Educational and Psychological Measurement.

[CR60] Monat A, Averill J, Lazarus R (1972). Anticipatory stress and coping reactions under various conditions of uncertainty. Journal of Personality and Social Psychology.

[CR61] Norton PJ (2005). A psychometric analysis of the Intolerance of Uncertainty Scale among four racial groups. Journal of Anxiety Disorders.

[CR62] Obschonka M, Silbereisen RK (2015). The effects of work-related demands associated with social and economic change on psychological well-being. Journal of Personnel Psychology.

[CR63] Rassin E, Muris P (2005). Indecisiveness and the interpretation of ambiguous situations. Personality and Individual Differences.

[CR64] Streiner DL, Norman GR, Cairney J (2008). Health measurement scales. A practical guide to their development and use.

[CR65] Tabachnick BG, Fidell LS (1996). *Using Multivariate Statistics*.

[CR66] Tanzer NK, Sim CQE (1999). Adapting instruments for use in multiple languages and cultures: A review of the ITC guidelines for test adaptations. European Journal of Psychological Assessment.

[CR67] Teale Sapach MJN, Carleton RN, Mulvogue MK, Weeks JW, Heimberg RG (2015). Cognitive constructs and social anxiety disorder: Beyond fearing negative evaluation. Cognitive Behaviour Therapy.

[CR68] Thibodeau MA, Carleton RN, McEvoy PM, Zvolensky MJ, Brandt CP, Boelen PA (2015). Developing scales measuring disorder-specific intolerance of uncertainty (DSIU): A new perspective on transdiagnostic. Journal of Anxiety Disorders.

[CR69] Thielsch C, Andor T, Ehring T (2015). Do metacognitions and intolerance of uncertainty predict worry in everyday life? An ecological momentary assessment study. Behavior Therapy.

[CR70] Thielsch C, Andor T, Ehring T (2015). Metacognitions, intolerance of uncertainty and worry: An investigation in adolescents. Personality and Individual Differences.

[CR71] Van de Vijver FJR, Hambleton RK (1996). Translating tests: Some practical guidelines. European Psychologist.

[CR72] Vandenberg RJ, Lance CE (2000). A review and synthesis of the measurement invariance literature: Suggestions, practices, and recommendations for organizational research. Organizational Research Methods.

[CR73] Zlomke KR, Hahn KS (2010). Cognitive emotion regulation strategies: Gender differences and associations to worry. Personality and Individual Differences.

