# Supplementary material for: Factorial validity and measurement invariance of the uncertainty response scale
Source: Psicol Reflex Crit. 2019 Dec 18;32:23. doi: 10.1186/s41155-019-0135-2 (PMC6967211; doi:10.1186/s41155-019-0135-2)
Supplement: Supplementary file 1 — Additional file 1. A. Descriptive statistics for the URS (Portuguese Version) [file 41155_2019_135_MOESM1_ESM.docx]

Supplementary Material A. Descriptive statistics for the URS (Portuguese Version)

|  |  | **EFA Sample (N=512)** | | | | | | | | | **CFA Sample (N=543)** | | | | | | **MG Sample (CFA2) (N=541)** | | | | | |  |
| --- | --- | --- | --- | --- | --- | --- | --- | --- | --- | --- | --- | --- | --- | --- | --- | --- | --- | --- | --- | --- | --- | --- | --- |
| **Items’**  **Number** | **Items’**  **Label** | | **Range** | **Min** | **Max** | **M** | **Md** | **SD** | **Sk** | **Ku** | | **M** | **MD** | **SD** | **Sk** | **Ku** | | **M** | **Md** | **SD** | **Sk** | **Ku** | |
| 1 | IEm2 | | 4 | 1 | 5 | 2.05 | 2.00 | 1.03 | .77 | -.07 | | 1.96 | 2.00 | 1.00 | .81 | -.21 | | 1.92 | 2.00 | 1.01 | .92 | -.06 | |
| 2 | ICog3 | | 4 | 1 | 5 | 3.29 | 4.00 | 1.36 | -.35 | -1.09 | | 3.26 | 3.00 | 1.34 | -.28 | -1.09 | | 3.3 | 3.00 | 1.32 | -.37 | -.97 | |
| 3 | ICog5 | | 4 | 1 | 5 | 3.96 | 4.00 | 1.04 | -.90 | .37 | | 4.03 | 4.00 | .94 | -.85 | .37 | | 3.91 | 4.00 | 1.00 | -.82 | .25 | |
| 4 | IEm6 | | 4 | 1 | 5 | 3.15 | 3.00 | 1.15 | -.18 | -.73 | | 3.06 | 3.00 | 1.14 | -.15 | -.80 | | 3.05 | 3.00 | 1.14 | -.05 | -.75 | |
| 5 | IEm7 | | 4 | 1 | 5 | 2.52 | 2.00 | 1.21 | .41 | -.80 | | 2.55 | 2.00 | 1.20 | .31 | -.91 | | 2.42 | 2.00 | 1.18 | .56 | -.56 | |
| 6 | ICog9 | | 4 | 1 | 5 | 3.9 | 4.00 | .86 | -.68 | .63 | | 3.92 | 4.00 | .84 | -.53 | .09 | | 3.84 | 4.00 | .92 | -.62 | .24 | |
| 7 | ICog10 | | 4 | 1 | 5 | 4.33 | 4.00 | .74 | -1.25 | 2.55 | | 4.29 | 4.00 | .74 | -.83 | .44 | | 4.3 | 4.00 | .77 | -1.24 | 2.37 | |
| 8 | IEm12 | | 4 | 1 | 5 | 2.71 | 3.00 | 1.13 | .27 | -.66 | | 2.64 | 3.00 | 1.13 | .25 | -.75 | | 2.7 | 3.00 | 1.12 | .30 | -.66 | |
| 9 | IEm13 | | 4 | 1 | 5 | 3.05 | 3.00 | 1.14 | -.02 | -.84 | | 2.99 | 3.00 | 1.14 | -.06 | -.77 | | 2.99 | 3.00 | 1.17 | .05 | -.87 | |
| 10 | IEm14 | | 4 | 1 | 5 | 3.52 | 4.00 | .98 | -.31 | -.41 | | 3.35 | 3.00 | 1.00 | -.32 | -.41 | | 3.39 | 4.00 | 1.01 | -.39 | -.35 | |
| 11 | IEm16 | | 4 | 1 | 5 | 2.7 | 3.00 | 1.21 | .21 | -.93 | | 2.57 | 2.00 | 1.17 | .29 | -.86 | | 2.56 | 2.00 | 1.19 | .35 | -.80 | |
| 12 | IDM18 | | 4 | 1 | 5 | 3.81 | 4.00 | .96 | -.51 | -.16 | | 3.74 | 4.00 | .94 | -.49 | -.11 | | 3.81 | 4.00 | .92 | -.52 | .09 | |
| 13 | IEm19 | | 4 | 1 | 5 | 2.93 | 3.00 | 1.14 | .10 | -.68 | | 2.85 | 3.00 | 1.16 | .08 | -.84 | | 2.81 | 3.00 | 1.15 | .19 | -.77 | |
| 14 | IDM21 | | 4 | 1 | 5 | 2.75 | 3.00 | 1.09 | .08 | -.70 | | 2.85 | 3.00 | 1.03 | -.13 | -.72 | | 2.82 | 3.00 | 1.07 | -.04 | -.68 | |
| 15 | IDM23 | | 4 | 1 | 5 | 4.51 | 5.00 | .89 | -2.14 | 4.55 | | 4.44 | 5.00 | .94 | -1.87 | 3.04 | | 4.5 | 5.00 | .87 | -2.05 | 4.10 | |
| 16 | IDM24 | | 4 | 1 | 5 | 3.27 | 3.00 | 1.24 | -.29 | -.87 | | 3.17 | 3.00 | 1.28 | -.23 | -.98 | | 3.18 | 3.00 | 1.26 | -.21 | -.93 | |
| 17 | IDM25 | | 4 | 1 | 5 | 4.33 | 4.00 | .81 | -1.39 | 2.54 | | 4.31 | 4.00 | .78 | -1.09 | 1.27 | | 4.38 | 4.50 | .73 | -1.15 | 1.65 | |
| 18 | IDM27 | | 4 | 1 | 5 | 4.15 | 4.00 | .91 | -1.16 | 1.47 | | 4.13 | 4.00 | .84 | -.76 | .40 | | 4.08 | 4.00 | .85 | -.75 | .39 | |
| 19 | ICog28 | | 4 | 1 | 5 | 4.22 | 4.00 | .78 | -.94 | 1.00 | | 4.24 | 4.00 | .74 | -.83 | .90 | | 4.22 | 4.00 | .74 | -.66 | .19 | |
| 20 | ICog29 | | 4 | 1 | 5 | 3.76 | 4.00 | .97 | -.46 | -.35 | | 3.71 | 4.00 | .98 | -.47 | -.25 | | 3.71 | 4.00 | .95 | -.48 | -.26 | |
| 21 | ICog30 | | 4 | 1 | 5 | 3.64 | 4.00 | .87 | -.39 | .08 | | 3.52 | 4.00 | .87 | -.22 | -.26 | | 3.55 | 4.00 | .82 | -.26 | .14 | |
| 22 | ICog32 | | 4 | 1 | 5 | 2.66 | 3.00 | 1.20 | .13 | -.91 | | 2.61 | 3.00 | 1.13 | .20 | -.78 | | 2.61 | 3.00 | 1.17 | .23 | -.81 | |
| 23 | IDM33 | | 4 | 1 | 5 | 4.17 | 4.00 | .85 | -1.03 | 1.17 | | 4.15 | 4.00 | .79 | -.70 | .26 | | 4.23 | 4.00 | .73 | -.87 | 1.44 | |
| 24 | IDM35 | | 4 | 1 | 5 | 4.09 | 4.00 | .82 | -.72 | .45 | | 4.09 | 4.00 | .79 | -.57 | .24 | | 4.16 | 4.00 | .77 | -.67 | .34 | |
| 25 | IDM36 | | 4 | 1 | 5 | 4.52 | 5.00 | .71 | -1.77 | 4.39 | | 4.54 | 5.00 | .64 | -1.27 | 1.70 | | 4.55 | 5.00 | .62 | -1.47 | 3.59 | |
| 26 | ICog37 | | 4 | 1 | 5 | 4.24 | 4.00 | .76 | -.99 | 1.63 | | 4.25 | 4.00 | .75 | -.90 | 1.14 | | 4.27 | 4.00 | .72 | -.78 | .63 | |
| 27 | ICog39 | | 4 | 1 | 5 | 3.61 | 4.00 | .96 | -.31 | -.49 | | 3.53 | 4.00 | .97 | -.26 | -.34 | | 3.52 | 4.00 | .93 | -.18 | -.46 | |
| 28 | ICog40 | | 4 | 1 | 5 | 3.93 | 4.00 | .82 | -.62 | .47 | | 3.90 | 4.00 | .86 | -.63 | .35 | | 3.95 | 4.00 | .81 | -.49 | .01 | |
| 29 | ICog42 | | 4 | 1 | 5 | 4.29 | 4.00 | .73 | -.83 | .75 | | 4.20 | 4.00 | .79 | -.75 | .31 | | 4.24 | 4.00 | .76 | -.68 | -.01 | |
| 30 | ICog44 | | 4 | 1 | 5 | 4.15 | 4.00 | .69 | -.72 | 1.45 | | 4.12 | 4.00 | .72 | -.69 | 1.00 | | 4.15 | 4.00 | .69 | -.65 | 1.15 | |
| 31 | IEm45 | | 4 | 1 | 5 | 3.51 | 4.00 | .91 | -.24 | -.08 | | 3.44 | 4.00 | .96 | -.45 | -.14 | | 3.45 | 4.00 | .94 | -.34 | -.14 | |
| 32 | IDM48 | | 4 | 1 | 5 | 3.97 | 4.00 | .79 | -.56 | .55 | | 3.99 | 4.00 | .79 | -.51 | .15 | | 4.05 | 4.00 | .77 | -.52 | .07 | |
| 33 | IEm50 | | 4 | 1 | 5 | 2.83 | 3.00 | 1.01 | .04 | -.30 | | 2.78 | 3.00 | 1.03 | -.04 | -.66 | | 2.78 | 3.00 | 1.02 | .01 | -.36 | |
| 34 | IDM51 | | 4 | 1 | 5 | 4.41 | 5.00 | .70 | -1.31 | 2.78 | | 4.41 | 5.00 | .69 | -1.07 | 1.57 | | 4.46 | 5.00 | .68 | -1.42 | 3.17 | |
| 35 | IEm53 | | 4 | 1 | 5 | 2.87 | 3.00 | 1.01 | .16 | -.44 | | 2.85 | 3.00 | .98 | -.10 | -.63 | | 2.84 | 3.00 | .98 | .11 | -.39 | |
| 36 | IEm54 | | 4 | 1 | 5 | 3.44 | 3.50 | .93 | -.31 | -.20 | | 3.32 | 3.00 | .99 | -.34 | -.29 | | 3.33 | 3.00 | 1.00 | -.29 | -.42 | |
| 37 | IDM55 | | 4 | 1 | 5 | 4.08 | 4.00 | .84 | -.77 | .54 | | 4.09 | 4.00 | .84 | -.57 | -.14 | | 4.16 | 4.00 | .79 | -.78 | .63 | |
| 38 | IDM57 | | 4 | 1 | 5 | 3.86 | 4.00 | .87 | -.54 | .17 | | 3.83 | 4.00 | .90 | -.42 | -.15 | | 3.89 | 4.00 | .89 | -.48 | -.10 | |
| 39 | ICog58 | | 4 | 1 | 5 | 3.54 | 4.00 | .90 | -.33 | -.07 | | 3.51 | 4.00 | .90 | -.32 | -.07 | | 3.45 | 4.00 | .95 | -.40 | -.09 | |
| 40 | IDM60 | | 4 | 1 | 5 | 3.49 | 4.00 | 1.09 | -.46 | -.34 | | 3.41 | 3.00 | 1.06 | -.32 | -.38 | | 3.44 | 3.00 | 1.01 | -.23 | -.39 | |
| 41 | IEm61 | | 4 | 1 | 5 | 2.74 | 3.00 | 1.00 | .23 | -.27 | | 2.72 | 3.00 | 1.01 | .08 | -.43 | | 2.75 | 3.00 | 1.02 | .15 | -.46 | |
| 42 | IDM62 | | 4 | 1 | 5 | 2.93 | 3.00 | .97 | -.14 | -.14 | | 2.96 | 3.00 | .96 | -.05 | -.09 | | 2.98 | 3.00 | .97 | .00 | .00 | |
| 43 | ICog63 | | 4 | 1 | 5 | 3.87 | 4.00 | 1.01 | -.70 | .02 | | 3.83 | 4.00 | 1.05 | -.63 | -.28 | | 3.86 | 4.00 | 1.02 | -.66 | -.25 | |
| 44 | IEm64 | | 4 | 1 | 5 | 2.96 | 3.00 | 1.13 | -.08 | -.70 | | 2.85 | 3.00 | 1.18 | .03 | -.86 | | 2.85 | 3.00 | 1.15 | .13 | -.75 | |
| 45 | IDM66 | | 4 | 1 | 5 | 3.74 | 4.00 | .93 | -.52 | .07 | | 3.75 | 4.00 | .88 | -.45 | .05 | | 3.79 | 4.00 | .89 | -.45 | .13 | |
| 46 | IEm68 | | 4 | 1 | 5 | 3.14 | 3.00 | .94 | -.05 | -.25 | | 3.09 | 3.00 | .91 | -.23 | -.27 | | 3.1 | 3.00 | .93 | -.08 | -.34 | |
| 47 | ICog69 | | 4 | 1 | 5 | 3.39 | 3.00 | 1.02 | -.27 | -.46 | | 3.41 | 3.00 | 1.00 | -.35 | -.29 | | 3.35 | 3.00 | 1.04 | -.27 | -.50 | |
| 48 | ICog70 | | 4 | 1 | 5 | 3.52 | 4.00 | 1.15 | -.50 | -.57 | | 3.52 | 4.00 | 1.11 | -.49 | -.43 | | 3.42 | 4.00 | 1.17 | -.37 | -.71 | |

*Note.* Min= minimum; Max=maximum; M=mean; Md=Median; SD=standard deviation; Sk=Skewness; Ku=Kurtosis
