# Supplementary material for: Factorial validity and measurement invariance of the uncertainty response scale
Source: Psicol Reflex Crit. 2019 Dec 18;32:23. doi: 10.1186/s41155-019-0135-2 (PMC6967211; doi:10.1186/s41155-019-0135-2)
Supplement: Supplementary file 2 — Additional file 2. B. URS’s Exploratory Factor Analysis – eigenvalues, variance explained [file 41155_2019_135_MOESM2_ESM.docx]

Supplementary Material B. URS’s Exploratory Factor Analysis – eigenvalues, variance explained

| **Factor** | **Eigenvalues** | **Variance Explained** |
| --- | --- | --- |
| Emotional Uncertainty (1) | 8.42 | 19.2 |
| Cognitive Uncertainty (2) | 6.75 | 15.1 |
| Desire for Change (3) | 2.84 | 5.44 |
