# Supplementary material for: Factorial validity and measurement invariance of the uncertainty response scale
Source: Psicol Reflex Crit. 2019 Dec 18;32:23. doi: 10.1186/s41155-019-0135-2 (PMC6967211; doi:10.1186/s41155-019-0135-2)
Supplement: Supplementary file 3 — Additional file 3. C. EFA - PAF with Oblique rotation (.4) – URS Distribution with item loadings [file 41155_2019_135_MOESM3_ESM.docx]

Supplementary Material C. EFA - PAF with Oblique rotation (.4) – URS Distribution with item loadings

| Items' Number | Items' Label | Emotional Uncertainty | Cognitive Uncertainty | Desire for Change |
| --- | --- | --- | --- | --- |
| 11 | IEm16 | .74 |  |  |
| 13 | IEm19 | .73 |  |  |
| 9 | IEm13 | .69 |  |  |
| 35 | IEm53 | .68 |  |  |
| 5 | IEm7 | .67 |  |  |
| 4 | IEm6 | .62 |  |  |
| 10 | IEm14 | .60 |  |  |
| 1 | IEm2 | .54 |  |  |
| 44 | IEm64 | .54 |  |  |
| 8 | IEm12 | .53 |  |  |
| 41 | IEm61 | .53 |  |  |
| 31 | IEm45 | .51 |  |  |
| 36 | IEm54 | .51 |  |  |
| 46 | IEm68 | .46 |  |  |
| 7 | ICog10 |  | .63 |  |
| 28 | ICog40 |  | .63 |  |
| 19 | ICog28 |  | .62 |  |
| 30 | ICog44 |  | .62 |  |
| 26 | ICog37 |  | .58 |  |
| 27 | ICog39 |  | .58 |  |
| 6 | ICog9 |  | .57 |  |
| 39 | ICog58 |  | .52 |  |
| 47 | ICog69 |  | .52 |  |
| 29 | ICog42 |  | .48 |  |
| 3 | ICog5 |  | .46 |  |
| 20 | ICog29 |  | .46 |  |
| 43 | ICog63 |  | .46 |  |
| 37 | IDM55 |  |  | .78 |
| 12 | IDM18 |  |  | .66 |
| 38 | IDM57 |  |  | .66 |
| 23 | IDM33 |  |  | .64 |
| 40 | IDM60 |  |  | .60 |
| 34 | IDM51 |  |  | .57 |
| 24 | IDM35 |  |  | .55 |
| 42 | IDM62 |  |  | .55 |
| 16 | IDM24 |  |  | .54 |
| 25 | IDM36 |  |  | .53 |
| 17 | IDM25 |  |  | .45 |
| 14 | IDM21 |  |  | .44 |
| 15 | IDM23 |  |  | .43 |
| 45 | IDM66 |  |  | .41 |
