# Supplementary material for: Factorial validity and measurement invariance of the uncertainty response scale
Source: Psicol Reflex Crit. 2019 Dec 18;32:23. doi: 10.1186/s41155-019-0135-2 (PMC6967211; doi:10.1186/s41155-019-0135-2)
Supplement: Supplementary file 4 — Additional file 4. D. CFA1 - URS Distribution after EFA (Sample 2); standardized coefficients (Model A) [file 41155_2019_135_MOESM4_ESM.docx]

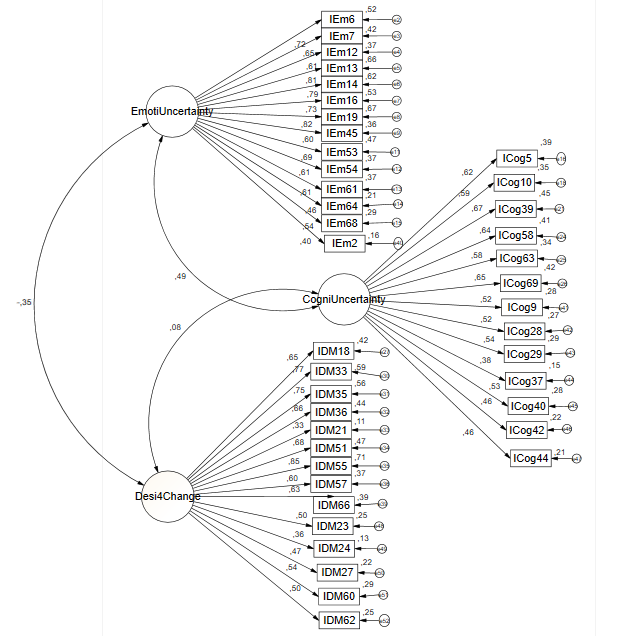


Supplementary Material D: CFA1 - URS Distribution after EFA (Sample 2); standardized coefficients (Model A)
