# Supplementary material for: Factorial validity and measurement invariance of the uncertainty response scale
Source: Psicol Reflex Crit. 2019 Dec 18;32:23. doi: 10.1186/s41155-019-0135-2 (PMC6967211; doi:10.1186/s41155-019-0135-2)
Supplement: Supplementary file 5 — Additional file 5. E. CFA1 - Final URS Distribution (Sample 2); standardized coefficients (Model B2) [file 41155_2019_135_MOESM5_ESM.docx]

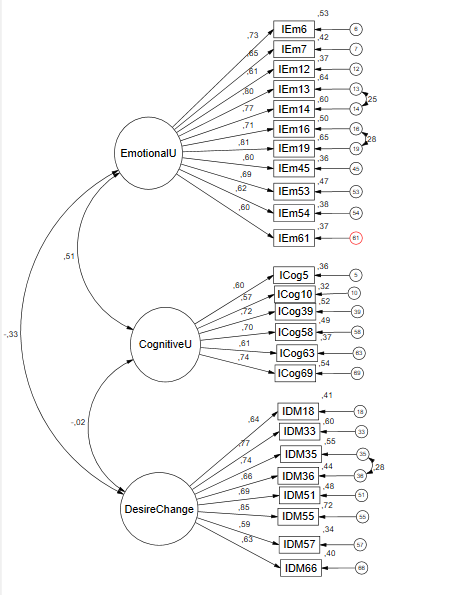


Supplementary Material E: CFA1 - Final URS Distribution (Sample 2); standardized coefficients (Model B2)
