# Supplementary material for: Factorial validity and measurement invariance of the uncertainty response scale
Source: Psicol Reflex Crit. 2019 Dec 18;32:23. doi: 10.1186/s41155-019-0135-2 (PMC6967211; doi:10.1186/s41155-019-0135-2)
Supplement: Supplementary file 7 — Additional file 7. G. Portuguese Adaptation of the Uncertainty Response Scale (48 items) [file 41155_2019_135_MOESM7_ESM.docx]

Supplementary Material G. Portuguese Adaptation of the Uncertainty Response Scale (48 items)

| **Factor 1 (Emotional Uncertainty)** | |
| --- | --- |
| 1. I tend to give up easily when I don't clearly understand a situation. | 1. Tenho tendência a desistir com facilidade quando não compreendo claramente uma situação. |
| **4. Sudden changes make me feel upset.** | **4. As mudanças demasiado súbitas põem-me aflito (a).** |
| **5. When making a decision, I am deterred by the fear of making a mistake.** | **5. Quando tenho de tomar uma decisão, quase paraliso pelo medo de cometer um erro.** |
| **8. When the future is uncertain, I generally expect the worst to happen.** | **8. Quando o futuro é incerto, geralmente acho que vai acontecer o pior.** |
| **9. Facing uncertainty is a nerve-wracking experience.** | **9. Deparar-me com a incerteza é uma experiência que me “dá cabo dos nervos”.** |
| **10. I get worried when a situation is uncertain.** | **10. Fico preocupado(a) quando não tenho certezas acerca de uma situação.** |
| **11. Thinking about uncertainty makes me feel depressed.** | **11. Pensar acerca da incerteza faz-me sentir deprimido(a).** |
| **13. Uncertainty frightens me.** | **13. A incerteza assusta-me.** |
| **31. When I can't clearly discern situations, I get apprehensive.** | **31. Quando não consigo perceber claramente as situações, fico apreensivo(a).** |
| 33. When I'm not certain about someone's intentions towards me, I often become upset or angry. | 33. Quando não tenho a certeza das intenções de alguém em relação a mim, geralmente fico aborrecido(a) ou zangado(a). |
| **35. When uncertain about what to do next, I tend to feel lost.** | **35. Quando estou na dúvida acerca do que fazer a seguir, tenho tendência a sentir-me perdido(a).** |
| **36. I feel anxious when things are changing.** | **36. Sinto-me ansioso(a) quando as coisas estão a mudar.** |
| **41. When a situation is unclear, it makes me feel angry.** | **41. Quando uma situação não é clara, sinto-me zangado(a).** |
| 44. I really get anxious if I don't know what someone thinks about me. | 44. Fico mesmo ansioso(a) quando não sei o que alguém pensa de mim. |
| 46. I am hesitant when it comes to making changes. | 46. Hesito quando tenho de mudar algo na minha vida. |
| **Factor 3 (Cognitive Uncertainty)** | |
| 2. When I go shopping, I like to have a list exactly of what I need. | 2. Quando vou às compras, gosto de ter uma lista com tudo o que preciso. |
| **3. I feel better about myself when I know that I have done all I can to accurately plan my future.** | **3. Sinto-me melhor quando sei que fiz tudo o que podia para planear cuidadosamente o meu futuro.** |
| 6. When uncertain, I act very cautiously until I have more information about the situation. | 6. Quando estou na dúvida, costumo agir de modo muito cauteloso até ter mais informação acerca da situação. |
| **7. I like to have things under control.** | **7. Gosto de ter as coisas sob controlo.** |
| 19. When I feel uncertain about something, I try to rationally weigh up all the information I have. | 19. Quando não tenho a certeza acerca de alguma coisa, tento analisar racionalmente toda a informação de que disponho. |
| 20. Before making any changes, I need to think things over thoroughly. | 20. Preciso de pensar muito nas coisas antes de fazer alguma mudança na minha vida. |
| 21. I prefer to stick to tried and tested ways of doing things. | 21. Prefiro fazer as coisas de um modo que já conheço e que funcionou no passado com sucesso. |
| 22. I like to have my weekends planned in advance. | 22. Gosto de planear os meus fins-de-semana com antecedência. |
| 26. When I feel a situation is unclear, I try to do my best to resolve it. | 26. Quando sinto que uma situação não é clara, tento dar o meu melhor para a resolver. |
| **27. I like to know exactly what I'm going to do next.** | **27. Gosto de saber exatamente aquilo que vou fazer a seguir.** |
| 28. When facing an uncertain situation, I tend to prepare as much as possible, and then hope for the best. | 28. Quando me deparo com uma situação incerta, tenho tendência a preparar-me o melhor possível e, depois, espero que tudo corra pelo melhor. |
| 29. I feel relieved when an ambiguous situation suddenly becomes clear. | 29. Sinto-me aliviado(a) quando uma situação ambígua de repente se torna clara. |
| 30. When I feel uncertain, I try to take decisive steps to clarify the situation. | 30. Quando me sinto na dúvida, tento fazer alguma coisa para clarificar a situação. |
| **39. I try to have my life and career clearly mapped out.** | **39. Tento ter a minha vida e carreira claramente planeadas.** |
| **43. I like things to be ordered and in place, both at work and at home.** | **43. Gosto que as coisas estejam organizadas e arrumadas no seu lugar, tanto no trabalho como em casa.** |
| **47. I like to plan ahead in detail rather than leaving things to chance.** | **47. Gosto de fazer planos detalhados e com antecedência em vez de deixar as coisas ao acaso.** |
| 48. Before I buy something, I have to view every sample I can find. | 48. Antes de comprar alguma coisa, tento ver todos os produtos do género que consiga encontrar. |
| **Factor 2 (Desire for change)** | |
| **12. I find the prospect of change exciting and stimulating.** | **12. Para mim, a possibilidade de mudanças na minha vida é entusiasmante e estimulante.** |
| 14. There is something exciting about being kept in suspense. | 14. Ficar em suspense tem algo de entusiasmante. |
| 15. The idea of taking a trip to a new country fascinates me. | 15. Fascina-me a ideia de fazer uma viagem a um país diferente. |
| 16. I like going on holidays with nothing planned in advance. | 16. Gosto de ir de férias sem planos prévios. |
| 17. I think you have to be flexible to work effectively. | 17. Considero que temos de ser flexíveis para trabalhar de modo eficaz. |
| 18. Taking chances is part of life. | 18. Correr riscos faz parte da vida. |
| **23. I feel curious about new experiences.** | **23. Sinto-me curioso(a) face a novas experiências.** |
| **24. I like to think of a new experience in terms of a challenge.** | **25. Gosto de encarar uma nova experiência como um desafio.** |
| **25. A new experience is an occasion to learn something new.** | **25. Uma nova experiência é uma oportunidade para aprender algo de novo.** |
| 32. I enjoy finding new ways of working out problems. | 32. Gosto de encontrar novas formas para resolver problemas. |
| **34. New experiences can be useful.** | **34. Viver novas experiências pode ser útil.** |
| **37. New experiences excite me.** | **37. Viver novas experiências entusiasma-me.** |
| **38. I think variety is the spice of life.** | **38. Para mim, a variedade é o que “apimenta” a vida.** |
| 40. I think a mid-life career change is an exciting idea. | 40. Acho excitante a ideia de mudanças de carreira durante a vida adulta. |
| 42. I enjoy unexpected events. | 42. Gosto de acontecimentos inesperados. |
| **48. I easily adapt to novelty.** | **48. Adapto-me facilmente à novidade.** |

*Note*. Items that are part of the Portuguese final version in Bold.
